# MiR-181a Negatively Regulates Claudin-3 to Facilitate *Lateolabrax maculatus* Iridovirus Replication in *Lateolabrax maculatus* Astroglia Cells

**DOI:** 10.3390/v16101589

**Published:** 2024-10-09

**Authors:** Yanping Ma, Jingjing Xu, Le Hao, Gang Wang, Wen Huang, Zhenxing Liu

**Affiliations:** 1 Institute of Animal Health, Guangdong Academy of Agricultural Sciences, Guangzhou 510640, China; mayanping@gdaas.cn (Y.M.); 15626199882@163.com (J.X.); haole@gdaas.cn (L.H.); wanggang@gdaas.cn (G.W.); 2 Collaborative Innovation Center of Guangdong Academy of Agricultural Sciences, Guangzhou 510640, China; huangwen@gdaas.cn; 3 Key Laboratory of Livestock Disease Prevention of Guangdong Province, Guangzhou 510640, China; 4 Institute of Animal Science, Guangdong Academy of Agricultural Sciences, Guangzhou 510640, China

**Keywords:** *Lateolabrax maculatus* iridovirus, Claudin-3, MiR-181a, virus replication

## Abstract

*Lateolabrax maculatus* iridovirus (LMIV) is a variant strain of red sea bream iridovirus (RSIV), causing serious economic losses in aquaculture. Claudins (CLDNs) are major components of tight junctions (TJs) forming an important line of defense against pathogens. Our pilot miRNA-mRNA joint analysis indicated the degradation of CLDN3, as well as its interaction with miR-181a during LMIV infection. To elucidate the miR-181a/CLDN3/LMIV interactions, in vitro assays were carried out on LMB-L cells. We first confirmed that LMIV infection could decrease the expression of CLDN3, accompanied by the enhancement of permeability, suggesting the dysfunction of TJs. Contrary to the inhibition of CLDN3, the activation of miR-181a was proved, presenting a negative correlation between miR-181a and CLDN3 (Pearson *r* = −0.773 and *p* < 0.01). In addition, the influence of CLDN3 on LMIV replication was analyzed by knockdown and over-expression of CLDN3. When CLDN3 was silenced in LMB-L cells with siCLDN3-623 at 9 days post transfection (dpt), LMIV copies and titers were significantly up-regulated by 1.59-fold and 13.87-fold, respectively. By contrast, LMIV replication in LMB-L cells was reduced by 60% and 71%, post transfection with pcDNA3.1-CLDN3 over-expressed plasmid at 6 dpt and 9 dpt, respectively. Ultimately, the regulatory relationship between miR-181a and CLDN3 was further validated by dual luciferase reporter assays. Taking into account the above-described results, we proposed a “miR-181a/CLDN3/LMIV” regulatory relationship. This study provides a new insight for understanding the mechanism of LMIV replication.

## 1. Introduction

Iridoviruses are cytoplasmic DNA viruses with an icosahedral capsid approximately 120–350 nm in diameter, which contains circularly permutated, terminally redundant, double-stranded DNA genomes (ICTV 2022). The family Iridoviridae is divided into seven genera, among them, megalocytiviruses, represented by red sea bream iridovirus (RSIV), are highly lethal pathogens, threatening a wide range of marine and freshwater fish such as spotted sea bass Lateolabrax maculatus [1,2,3,4]. *L. maculatus* iridovirus (LMIV) is a variant RSIV strain identified in our previous study [5]. LMIV has circulated in spotted sea bass in China and caused serious economic losses, and phylogenetic analysis ascertained that LMIV had a 98.8% similarity with Large yellow croaker iridovirus (LYCIV) and a 94.8% similarity with Infectious spleen and kidney necrosis virus (ISKNV) [5]. LMIV-infected fish exhibited a dark body color, abnormal swimming, an enlarged spleen, and more than 80% mortality.

Tight junctions (TJs) are intercellular multi-protein complexes, building the barrier to protect against pathogen invasion [6]. Claudins are the major membrane-related tight junction proteins, with the same conformational characteristics containing four transmembrane domains and two extracellular loops [7,8]. The loss of claudins leads to the destruction of TJs, followed by the collapse of their selective permeability [6]. Mounting studies have shown that claudins may participate in virus invasion [9,10,11,12,13,14]. Claudin-3 (CLDN3), a member of the claudin family, correlates with infection by HIV [11], simian immunodeficiency virus (SIV) [15], porcine reproductive and respiratory syndrome virus (PRRSV) [9], avian influenza virus subtype H9N2 infection [10], and so on. Similar descriptions are also found in teleost. For instance, cyprinid herpesvirus 3 (CyHV3) infection disrupts the skin and intestinal barrier of common carp, causing the down-regulation of claudin-3C and other claudin proteins [16,17]. The underling mechanisms by which viruses regulate CLDNs have attracted increasing attention. In particular, microRNA (miRNA) opens up a new perspective to illustrate the regulatory relationship. MiRNA is a small noncoding RNA that impairs protein expression via base pairing with mRNA [18]. Viruses have acquired the ability to affect host miRNAs to benefit viral replication [19]. MiR-181a is widely found in various kinds of organisms, and has been studied as a regulatory miRNA with altered expression in various diseases [20]. Previous studies have shown that miR-181a played roles in the invasion and migration of cells [21], inflammatory responses [22], tumorigenesis [20], and the immune evasion of hepatitis B virus (HBV) [23]. In our pilot study, we performed an miRNA-mRNA joint analysis using samples from LMIV-infected LMB-L cells and noticed that LMIV infection reduced the expression of CLDN3 that was targeted by up-regulated cgr-miR-181a-5p (miR-181a). Thus, we speculated that there existed a regulatory mechanism, i.e., “miR-181a/CLDN3/LMIV”.

In the present work, the correlations between LMIV, CLDN3, and miR-181a were analyzed, followed by the validation of targeting relationship between miR-181a and CLDN3. The effect of LMIV-induced down-regulation of CLDN3 on TJ functions was evaluated by dextran permeability assays. Then, the influence of CLDN3 expression on LMIV replication was assessed under either CLDN3 over-expression or knockdown conditions. This work aimed to elucidate an LMIV replication mechanism associated with CLDN3 negatively regulated by miR-181a.

## 2. Materials and Methods

### 2.1. Virus and Cells

Spotted sea bass (*L. maculatus*) brain cells, LMB-L, were maintained in Leibovitz’s 15 (L-15) medium containing 10% fetal bovine serum (FBS, Gibco, New York, NY, USA) at 25 °C as we previously described [5]. HEK-293 cells were cultured in DMEM medium containing 10% FBS at 37 °C, 5% CO_2_. LMIV was isolated and identified as an atypical strain of RSIV in our previous study [5].

### 2.2. Expression Analysis of CLDN3 and miR-181a by qRT-PCR

The open reading frame (ORF) of the CLDN3 gene was cloned using the primer pairs CLDN3-F/CLDN3-R (Table 1) designed by Oligo 7.0 software, according to the gene sequence in GenBank database (No. URX57470.1). The encoded amino acid sequence was predicted with DNAstar software 7.1. The phylogenetic analysis and multiple sequence alignment based on deduced amino acid sequence of CLDN3 were performed by the neighbor-joining (NJ) method with 1000 bootstrap replicates using MEGA software 11.0., Protein tertiary structure was predicted online via the ExPASy website (https://swissmodel.expasy.org/, accessed on 1 August 2024). The mature miR-181a was cloned with the primer pairs miR181a-F/miR181a-R, according to the gene sequence in the miRBase database (No. MIMAT0023797).

To examine the expression profiles of miRNAs and CLDN3 during LMIV infection in LMB-L cells, the cells (4 × 10^5^ cells per plate) were transferred to 6-well plates and cultured in L-15 medium containing 10% FBS at 25 °C until the confluence reached 80%. Then, the cells were inoculated with LMIV, starting at a 1:10 dilution (10^7.125^ TCID_50_/mL), for 2 h to allow virus attachment and entry. The supernatant media was discarded, and the cells were washed 3 times with PBS. Fresh L-15 medium containing 2.5% FBS was added to maintain the cells. The cells were collected at 0, 1, 2, 3, 4, 5, 6, 9, and 12 days post infection, respectively, for examining the expression of miR-181a and CLDN3, as well as viral loads. Assessment of miR-181a and CLDN3 expression was carried out by qRT-PCR as follows. RNA was extracted by TRIzol Kit (ThermoFisher, Waltham, MA, USA). Then, cDNA was synthesized with the HiScript^®^ II Q RT Supermix Kit (Vazyme, Nanjing, China) and Mir-X miRNA First-Strand Synthesis Kit (TaKaRa, Dalian, China), respectively. The obtained cDNA served as templates for CLDN3 and miR-181a expression analysis by qRT-PCR according to the manufacturer’ s instructions (AceQ qPCR SYBR Green Master Mix, Vazyme and Mir-X miRNA qRT-PCR TB Green^®^ Kit, TaKaRa, Dalian, China), respectively. The primers are listed in Table 1. The CLDN3 qRT-PCR program was 95 °C for 5 min, followed by 40 cycles of 95 °C for 10 s and 60 °C for 30 s. The miR-181a qRT-PCR program was 95 °C for 10 s, followed by 40 cycles of 95 °C 5 s and 60 °C for 20 s. The amplification products were analyzed by CFX Connect Real-Time System (Bio-Rad, Hercules, CA, USA). Expression changes in CLDN3 and miR-181a were standardized against RNA polymerase II and the U6 gene, respectively, using the 2^−ΔΔCt^ method. Viral loads were determined by absolute quantification qPCR as presented in our previous study [5]. The correlations among LMIV copies, CLDN3 expression, and miR-181a abundance were analyzed using SPSS software 23.0, according to the previous study [24].

### 2.3. Over-Expression of CLDN3 in LMB-L Cells

CLDN3 over-expression plasmid was generated by inserting CLDN3 ORF-containing signal peptide into plasmid pcDNA3.1 (Invitrogen, Carlsbad, CA, USA). In detail, CLDN3 ORF was amplified by the primer pairs CLDN3 ORF-F/CLDN3 ORF-R using the above-cloned CLDN3 as a template. The product was purified and ligated to the pcDNA3.1 vector through EcoRI and XhoI restriction sites. The recombinant plasmid, named pcDNA3.1-CLDN3, was confirmed by sequencing.

LMB-L cells (4 × 10^5^ cells per well) were seeded into 6-well plates and cultured in L-15 medium containing 10% FBS at 25 °C until the confluence reached 80%, then the pcDNA3.1-CLDN3 over-expression plasmid (2.5 μg) was transfected into cultured cells with lipofectamine^TM^ 3000 (Invitrogen, USA). CLDN3 mRNA expressions were determined at different times post transfection. And CLDN3 protein expression was determined using mouse against CLDN3 antibody by indirect immunofluorescence and Western blotting.

After 48 h, the transfected cells were inoculated with LMIV for 2 h at a final titer of 10^7.125^ TCID_50_/mL to allow viral attachment and entry. Then, the cells were washed 3 times with PBS followed by adding fresh L-15 medium containing 2.5% FBS until further analysis. Cells transfected with empty vector pcDNA3.1 and untransfected cells served as controls.

### 2.4. SiRNA Transfection and LMIV Infection

SiCLDN3-176- and siCLDN3-623-targeting *L. maculatus* CLDN3 mRNA, as well as siRNA-control (siCtrl)-containing random nucleotides unable to bind to *L. maculatus* CLDN3 mRNA were designed and synthesized by Sangon company (Shanghai, China). The corresponding sequences are listed in Table 2. The LMB-L cells (4 × 10^5^ cells per well) were sub-cultured into 6-well plates and kept in L-15 medium containing 10% FBS at 25 °C until the confluence reached 80%. Then, 40 nM siRNAs (siCLDN3-176, siCLDN3-623 and siCtrl) were transfected into LMB-L cells with Lipofectamine™ 3000 reagent (Invitrogen, USA), respectively. CLDN3 mRNA expressions were determined at different times post transfection. And CLDN3 protein expression were determined using mouse antibodies against CLDN3 antibody by indirect immunofluorescence and Western blotting.

After 24 h, the transfected cells were inoculated with LMIV as described above. Two hours after exposure to LMIV, the cells were washed 3 times with PBS and subsequently cultured in fresh L-15 medium containing 2.5% FBS for further analysis. Cells transfected with siCtrl and untransfected cells served as the controls.

### 2.5. Virus Titration

LMB-L cells cultured in 96-well plates (1 × 10^4^ cells/well) were transfected with CLDN3 over-expression plasmid (pcDNA3.1-CLDN3) and siRNA (siCLDN3-623), respectively. Forty-eight hours post over-expression or post knockdown, a 10-fold serial dilution of LMIV (10^8.125^ TCID_50_/mL) was made, and each dilution was inoculated onto cells in 8 replicate wells. LMB-L cells transfected with siCtrl and pcDNA3.1, respectively, were used as controls. CPEs were observed daily, and virus titers were calculated by the Reed–Muench method [25].

### 2.6. Permeability Assay

Permeability assay was carried out according to the previous study with some modification [26]. LMB-L cells (5 × 10^4^ cells/well) were cultured onto 0.4 μm pore size polycarbonate membrane tissue culture plate inserts (24 wells). FITC-Dextran (30 μg/mL) was added into the upper wells to evaluate the barrier function of tight junctions every day by measuring the fluorescence values. Until the relative fluorescence value (upper well/lower well) no longer decreased, the formation of the tight junction was stable. LMIV was serially diluted at 1:2, starting at 5 × 10^7^ TCID_50_/mL, then exposed to the apical surface for 24 h. Subsequently, 30 μg/mL FITC-Dextran (Sigma, St. Louis, MO, USA, Mw 150,000) was added in the upper wells of each insert for 30 min. Then, cultures in the upper well and lower well plate inserts were transported to 96-well black culture plates and immersed in fresh L-15, to measure fluorescence value by multiscan spectrum, respectively (ThermoFisher, Waltham, MA, USA).

### 2.7. MiRNA-mRNA Joint Analysis

LMB-L cells were inoculated with LMIV for 2 h to allow virus attachment and entry. The supernatant media was discarded, and the cells were washed 3 times with PBS. Fresh L-15 medium containing 2.5% FBS was added to maintain the cells. The cells were collected at 6 days post infection for mRNA sequencing and miRNA sequencing. Then, differentially expressed miRNAs and differentially expressed mRNAs were analyzed jointly by LC-Bio. The possible binding sites of miR-181a and CLDN3 were predicted by the RNAhybrid online software 2.1 (https://bibiserv.cebitec.uni-bielefeld.de/rnahybrid, accessed on 1 August 2024).

### 2.8. Dual-Luciferase Reporter Assays

The MiR-181a mimic and miR-negative were synthesized by Sangon company (Shanghai, China). The 3′-UTR of the CLDN3 gene was amplified by PCR with the primer pairs CLDN3 3′-UTR-F/CLDN3 3′-UTR-R (Table 1). The PCR product was purified and inserted into the dual-luciferase vector pmirGLO (Youbao, Changsha, China) at the XhoI/SalI sites, generating the pmirGLO-CLDN3-WT-3′-UTR plasmid. The pmiGLO-CLDN3-Mut-3′-UTR construction was identical to pmirGLO-CLDN3-WT-3′-UTR, except that the sequences of the 3′-UTR of CLDN3 binding to the miR-181a seed sequence were complementarily mutated by overlap PCR.

To verify the binding of miR-181a to CLDN3 3′-UTRs, HEK293 cells (5 × 10^4^ cells per well) were seeded into 96-well plates and cultured in DMEM medium containing 10% FBS until the confluence reached 80% at 37 °C, 5% CO_2_. The cells were co-transfected with 100 ng of plasmid (one of pmirGLO-CLDN3-WT-3′-UTR, pmirGLO-CLDN3-Mut-3′-UTR) and 100 nM of the synthesized miRNA (one of miR-181a mimic, miR negative) with Lipofectamine™ 3000 (Invitrogen, Carlsbad, CA, USA) according to the manufacturer’s instructions. MiR-negative plus pmirGLO-CLDN3 WT-3′-UTR and miR-negative plus pmirGLO-CLDN3 Mut-3′-UTR were regarded as controls. Twenty-four hours post transfection, the cells were lysed, firefly luciferase activity and Renilla luciferase activity were measured using a dual-luciferase reporter assay kit (Promega, Madison, WI, USA).

### 2.9. Statistical Analysis

All experiments in this study were performed in triplicate independently. Unpaired Student’ s *t*-test was analyzed for the correlations among LMIV copies, CLDN3 expression, and miR-181a abundance. Other statistical analyses were calculated using one-way ANOVA in SPSS software 23.0. The data are presented as mean ± standard deviation (SD). *p*-values < 0.05 were considered to be statistically significant. *p*-values < 0.01 were considered to be highly significant. Significance is depicted as not significant (ns), * *p* < 0.05, and ** *p* < 0.01.

## 3. Results

### 3.1. The Molecular Features of CLDN3 and miR-181a

**Figure 1:**
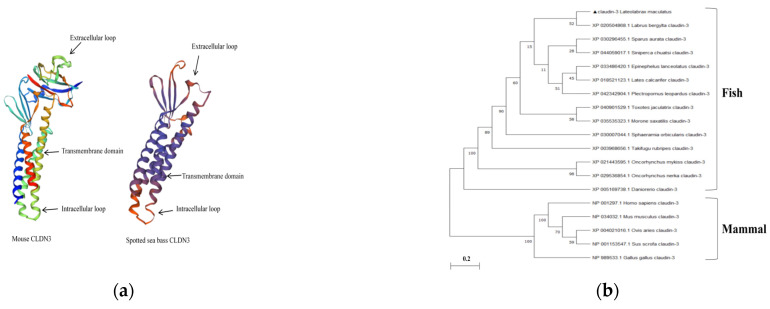
Analysis of spotted sea bass CLDN3 protein sequences. (**a**) Structural comparison of CLDN3 was performed between spotted sea bass and mouse by online swiss-model. Typical domains are indicated by arrows. (**b**) Phylogenetic trees of full-length CLDN3s were constructed using the neighbor-joining procedure in MEGA software 11.0. The numbers on the forks indicate the percentage of bootstrap support from 1000 replicates. Genbank accession numbers are given in front of species names. Spotted sea bass CLDN3 is highlighted by the blank triangle.

The spotted sea bass CLDN3 cDNA contains 648 bp nucleotides, encoding 216 amino acids with a putative molecular mass of 23.14 kDa. The domain architecture of the spotted sea bass CLDN3 protein was further predicted, indicating that the classical extracellular loop and intracellular loop were found in spotted sea bass CLDN3. Generally speaking, spotted sea bass CLDN3 shared a similar tertiary structure with that of mouse (PDB Hit: 6ake.1) by homology modeling (Figure 1a). Moreover, the CLDN3-based phylogenetic tree revealed that spotted sea bass clustered into one branch with other fish, indicating a closer evolutionary relationship (Figure 1b). The sequence comparison and homology modeling analysis suggested that spotted sea bass CLDN3 possessed a conserved and crucial function like other fish and mammals. The spotted sea bass mature miR-181a has a 23 bp length and has 100% similarity with the miR-181a sequence in the miRBase database (No. MIMAT0023797).

### 3.2. Correlation Existed among LMIV Copies, Expression of CLDN3, and miR-181a

**Figure 2:**
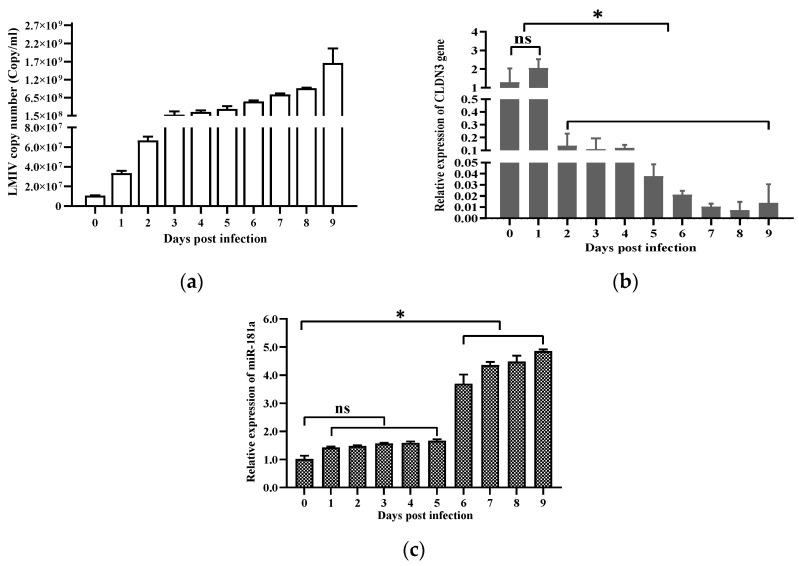
Expression of CLDN3, miR-181a, and replication of LMIV. CLDN3 expression was down-regulated and miR-181a expression was up-regulated in LMB-L cells post LMIV infection. The non-infected cells (0 d) served as negative controls. Total RNA, including mRNA and microRNA, was collected for expression assessment of CLDN3 and miR-181a at different time points; meanwhile, the DNA of LMIV-infected cells was used for the calculation of LMIV copies. (**a**) Virus copy numbers increased in LMB-L cells after exposure to LMIV. (**b**,**c**) The relative expression of CLDN3 (**b**) and miR-181a (**c**) was determined in LMIV-infected LMB-L cells, normalized to RNA-polymerase II or the U6 gene, respectively, exhibiting an inverse trend between the miR-181a levels and the expression of CLDN3. The data are presented as a mean ± SD (*n* = 9); ns, not significant, * *p* < 0.05.

In order to investigate whether CLDN3 and miR-181a could be regulated by LMIV infection, correlation analysis among them was determined by Spearman’ s correlation. It was found that, as the time increased, the rises in LMIV copies were detected (Figure 2a). Throughout the infection course, consistent inhibition of CLDN3 expression and enhancement of miR-181a expression were determined in LMIV-infected LMB-L cells (Figure 2b,c). In addition, Spearman’ s correlation analysis demonstrated that miR-181a expression levels negatively correlated with CLDN3 expression levels (Pearson *r* = −0.565 and *p* < 0.05). Similarly, CLDN3 expression had a significantly negative correlation with LMIV copies (Pearson *r* = −0.773 and *p* < 0.01). By contrast, miR-181a expression levels positively correlated with LMIV copy numbers (Pearson *r* = −0.834 and *p* < 0.05).

### 3.3. Over-Expression of CLDN3 Inhibited LMIV Replication

**Figure 3:**
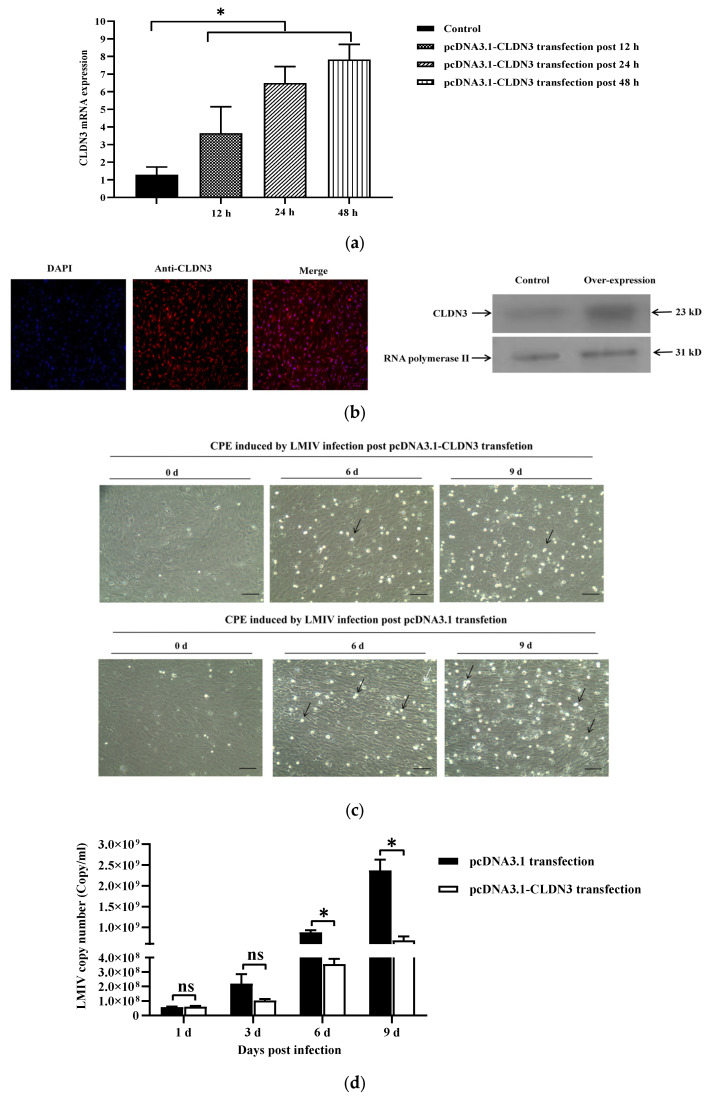
Over-expression of CLDN3 inhibited LMIV replication in LMB-L cells. (**a**) The relative expression of CLDN3 in pcDNA3.1-CLDN3-transfected cells, normalized to that in pcDNA3.1-transfected cells, was assessed at 12 h.p.t, 24 h.p.t, and 48 h.p.t. (**b**) Over-expression analysis of CLDN3 in pcDNA3.1-CLDN3 plasmid-transfected cells by indirect immunofluorescence and Western blotting (Scale bar = 100 μm). (**c**) CPE induced by LMIV infection was mitigated by CLDN3 over-expression, with cell morphology and cytoplasmic vacuolization changed. Scale bars represent 100 μm; Black arrow indicates infected-detached cells, White arrow indicates infeted-loose cells. (**d**) After 48 h of transfection with pcDNA3.1-CLDN3 or pcDNA3.1 (control), transfected cells were exposed to LMIV and viral loads were quantified by the absolute quantitative qPCR method at 1, 3, 6, and 9 d.p.i. Over-expression of CLDN3 significantly inhibited LMIV replication in LMB-L cells at 6 d.p.i. and 9 d.p.i. The values are shown as means ± SD (*n* = 9) and analyzed by one-way ANOVA test; ns, not significant, * *p* < 0.05.

In order to investigate the influence of CLDN3 over-expression on LMIV replication in LMB-L cells, LMB-L cells were transfected with the pcDNA3.1-CLDN3 plasmid. Then, the significant up-regulation of CLDN3 mRNAs was determined at 12 h, 24 h, and 48 h post transfection, and CLDN3 mRNA expression obtained eight-fold up-regulation (Figure 3a). In addition, mouse anti-CLDN3 antibody and mouse anti-RNA polymerase II antibody were prepared (Figure S1). Using these antibodies, transfection of the pcDNA3.1-CLDN3 plasmid led to up-regulation of CLDN3 in LMB-L cells, respectively, which was ascertained by indirect immunofluorescence and Western blotting (Figure 3b). After the verification of over-expression efficiency, cells transfected with pcDNA3.1-CLDN3 for 48 h were chosen for the subsequent LMIV exposure experiment. LMIV-infected cells revealed typical CPE, including cell shrinkage, rounding, and cytoplasmic vacuolization, while the severity of CPE was significantly ameliorated in pcDNA3.1-CLDN3-transfected cells (Figure 3c). Correspondingly, absolute quantification PCR confirmed that CLDN3 over-expression reduced LMIV loads from 8.78 × 10^8^ copies/mL to 3.55 × 10^8^ copies/mL at 6 d.p.i. and from 6.83 × 10^8^ copies/mL to 2.37 × 10^9^ copies/mL at 9 d.p.i. (*p* < 0.05) (Figure 3d). This demonstrated that the over-expression of CLDN3 inhibited LMIV replication in vitro.

### 3.4. Knockdown of CLDN3 Enhanced LMIV Replication

**Figure 4:**
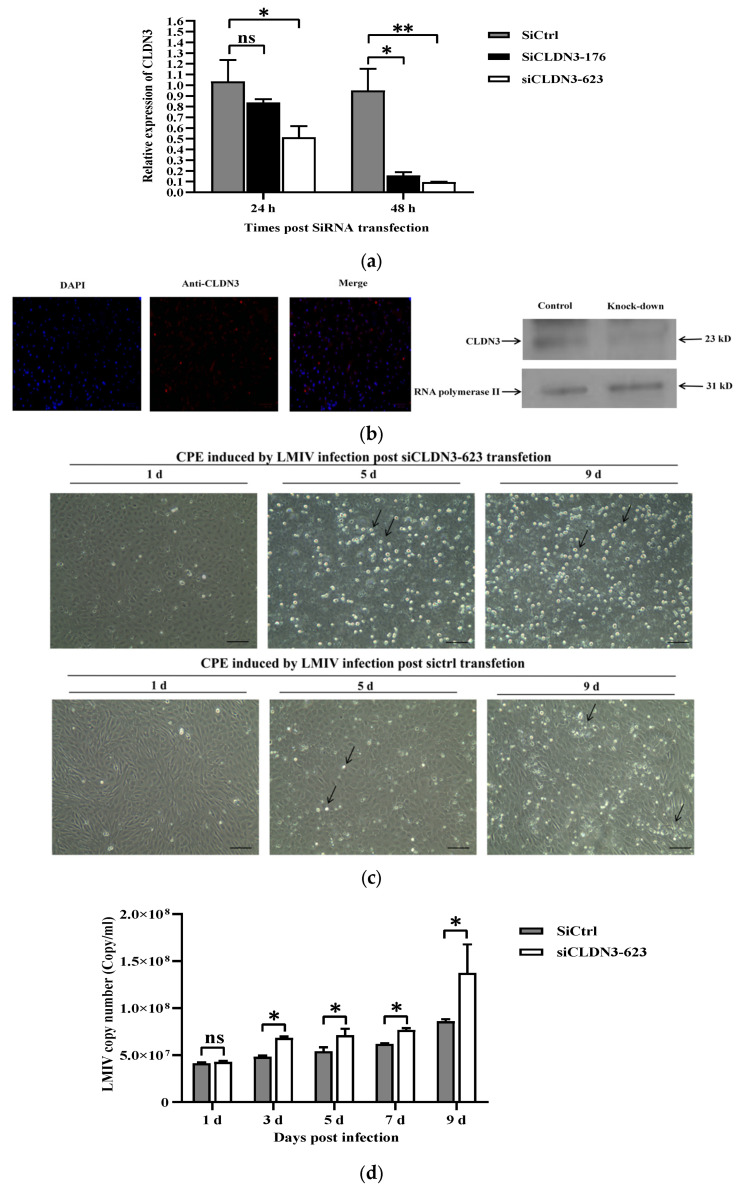
Knockdown of CLDN3 using siRNA facilitated LMIV replication in LMB-L cells. (**a**) The relative expression of CLDN3 was detected post siCLDN3-176 and siCLDN3-623 transfection in LMB-L cells, normalized to siCtrl-transfected cells. SiCLDN3-623 led to lower CLDN3 expression levels, compared with siCLDN3-176. (**b**) Knockdown analysis of CLDN3 in siRNA-transfected cells by indirect immunofluorescence and Western blotting (Scale bar = 100 μm). (**c**) CPE induced by LMIV infection was aggravated by siCLDN3-623 transfection, with cell morphology and cytoplasmic vacuolization changed. Scale bars represent 100 μm; Black arrow, infected-detached cells. (**d**) LMIV copies were quantified post knockdown of CLDN3. Forty-eight hours after transfection with siCLDN3-623, the cells were subjected to LMIV infection, followed by virus quantification with qPCR. This showed that siCLDN3-623 transfection significantly improved LMIV copies in LMB-L cells at 3, 5, 7, and 9 d.p.i. The data are presented as mean ± SD; ns, not significant, * *p* < 0.05, ** *p* < 0.01.

For the knockdown of CLDN3, two siRNAs (siCLDN3-176 and siCLDN3-623) were designed to seek the preferable one. Following siRNA transfection, CLDN3 was quantified by qRT-PCR. It was found that significant down-regulation of CLDN3 was available using either siCLDN3-176 or siCLDN3-623, compared with the non-targeting siRNA (siCtrl) (Figure 4a). The more efficient siCLDN3-623, generating a 10-fold decrease in CLDN3 level, was chosen for the knockdown of CLDN3. Furthermore, transfection of siCLDN3-623 led to down-regulation of CLDN3 in LMB-L cells, respectively, which was ascertained by indirect immunofluorescence and Western blotting (Figure 4b). LMIV-infected cells revealed typical CPE, including cell shrinkage, rounding, and cytoplasmic vacuolization through light microscope observation at 5 d and 9 d, while the severity of CPE was significantly exacerbated in siCLDN3-623-transfected cells (Figure 4c). Knockdown of CLDN3 facilitated LMIV replication, as shown in Figure 4d, and the viral loads rose from 4.81 × 10^7^ copies/mL to 6.81 × 10^7^ copies/mL at 3 d.p.i., from 5.40 × 10^7^ copies/mL to 7.10 × 10^7^ copies/mL at 5 d.p.i., from 6.18 × 10^7^ copies/mL to 7.65 × 10^7^ copies/mL at 7 d.p.i. and from 8.60 × 10^7^ copies/mL to 1.37 × 10^8^ copies/mL at 9 d.p.i. This demonstrates that knockdown of CLDN3 enhanced LMIV replication in vitro.

### 3.5. CLDN3 Regulated the Virus Titer of LMIV

Forty-eight hours post CLDN3 knockdown or CLDN3 over-expression in LMB-L cells, as in the above-mentioned transfection, LMIV (10^8.125^ TCID_50_/mL) was 10-fold serially diluted, then inoculated onto the transfected cells to calculate viral titers. We found that the knockdown of CLDN3 significantly increased the virus titer up to 10^9.267^ TCID_50_/mL (*p* < 0.05), while CLDN3 over-expression presented an opposite result with the virus titer reduced to 10^7.292^ TCID_50_/mL (*p* < 0.01).

### 3.6. Permeability Enhancement of LMB-L Cells Post LMIV Infection

**Figure 5:**
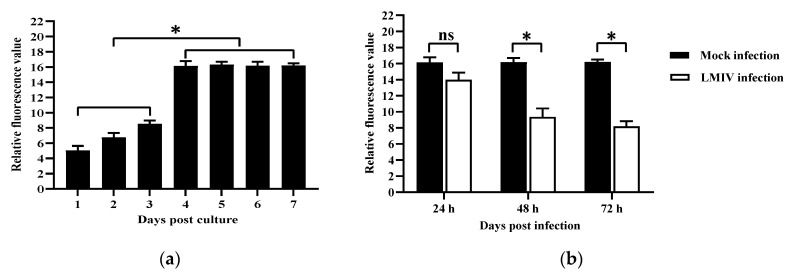
Assessment of permeability by FITC-Dextran. (**a**) The fluorescence ratios of the upper wells to the lower wells was measured, revealing that the function of the tight junction was stable after 4 days of culture. (**b**) LMIV infection increased the permeability of LMB-L cells. The relative fluorescence values were determined at 24 h, 48 h, and 72 h post LMIV infection, respectively; ns, not significant, * *p* < 0.05.

LMB-L cells were cultured onto polycarbonate membrane tissue culture plate inserts (24 wells), then FITC-Dextran was added into the upper wells to evaluate the barrier function of the tight junction. As shown in Figure 5a, relative fluorescence value no longer decreased from the 4th day, indicating the tight junction in the cultured cells tended to be stable. Therefore, after 4 days of culture, the cells in the upper wells were inoculated with LMIV for 24 h, 48 h, and 72 h, respectively. Then, FITC-Dextrans was added into the upper wells to assess cell permeability. The fluorescence values of the upper and lower wells were measured, as shown in Figure 5b, and the relative fluorescence value in the LMIV-infected cells significantly decreased at 48 h and 72 h post infection (*p* < 0.05). These results suggested that LMIV infection enhanced the permeability of LMB-L cells and disrupted the tight junction integrity of LMB-L cells.

### 3.7. MiRNA and mRNA Joint Analysis

**Figure 6:**
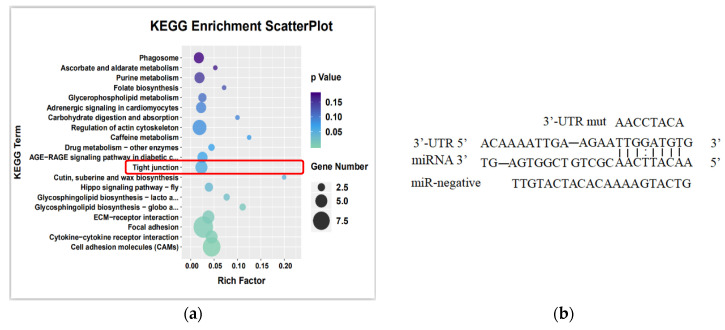
The analysis of correlation between miR-181a and CLDN3. (**a**) The KEGG enrichment analysis of mRNA sequencing after LMIV infection; The red box indicates Tight junction signaling pathway. (**b**) Schematic diagrams of microRNA target prediction show the miR-181a binding site in the 3′-UTR of CLDN3.

The mRNA sequencing and miRNA sequencing were performed with LMIV-infected LMB-L cells at 6 d.p.i. Twenty differentially expressed miRNAs and 711 differentially expressed mRNAs were found. And KEGG analysis showed that differentially expressed genes were significantly enriched in the tight junction signaling pathway (Figure 6a). Then, the possible binding sites of the differentially expressed miRNAs and differentially expressed tight junction-related genes were predicted by RNAhybrid, as shown in Figure 6b; the 3′-UTR of CLDN3 comprised a binding site for miR-181a, in which the sequence of CLDN3 3′-UTR was complementary to the seed region of miR-181a.

### 3.8. MiR-181a Suppressed the Expression of CLDN3

**Figure 7:**
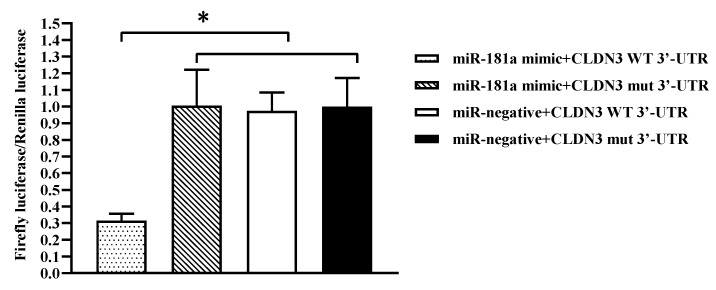
The analysis of interaction between miR-181a and the 3′-UTR of CLDN3 by dual-luciferase reporter assays. HEK293 cells were co-transfected with miR181a mimic, miR-negative, and different plasmids (pmirGLO-CLDN3-WT-3′-UTR and pmirGLO-CLDN3-Mut-3′-UTR). Firefly luciferase/Renilla luciferase activity was measured after 24 h of transfection, and significantly reduced relative luciferase activity was observed in cells transfected with miR-181a mimic plus pmirGLO-CLDN3-WT-3′-UTR. The values are shown as means ± SD (*n* = 9). The significance levels were set as * *p* < 0.05.

In order to verify the correlation between miR-181a and CLDN3, the reporter plasmids were constructed, harvesting CLDN3 WT 3′-UTR and CLDN3 mut 3′-UTR, respectively. Different double combinations (one from miR-181a mimic and miR-negative and the other from the reporter plasmids) were transfected into HEK293 cells. Relative luciferase activity was significantly reduced in cells transfected with miR-181a mimic plus CLDN3 WT 3′-UTR. Conversely, luciferase activity remained stable in HEK293 cells co-transfected with the reporter plasmids and miR-negative. Meanwhile, no significant changes in luciferase activity were detected in HEK293 cells transfected with miR-181a mimic plus CLDN3 mut 3′-UTR, compared with the control cells (Figure 7).

## 4. Discussion

Megalocytivirus belongs to the Iridoviridae family and has an icosahedral capsid approximately 140–200 nm in diameter, which contains circularly permutated, terminally redundant, double-stranded DNA genomes. Many iridoviruses have been reported to exploit the host cellular machinery to accomplish their life cycle. Infectious spleen and kidney necrosis virus (ISKNV) infection needs the actin cytoskeleton to facilitate its replication [27,28]. Singapore grouper iridovirus (SGIV) infection enhances virus multiplication by decreasing intestine integrity and tight junction protein expression [29].

Accompanied by the multiplication of LMIV, the expression of CLDN3 was dramatically reduced. Correspondingly, knockdown of CLDN3 promoted the LMIV proliferation, and the rising trend in virus replication could be reversed by over-expression of CLDN3. The above-mentioned results indicate that CLDN3 interacted with LMIV. We could understand the interaction mechanism from the assembly of CLDNs into tight junctions (TJs). TJs form physical barriers such as the BBB, intestinal barrier, skin barrier, and cultured cell junction barrier to resist pathogens and maintain homeostasis. Viruses have evolved specific mechanisms to break through these barriers. CLDNs, as major components of TJs, unsurprisingly intervene in the course of virus invasion [17,30], especially CLDN3, which is regarded as a key component determining the integrity of the cell junction barrier [6]. Two main types of interactions between CLDNs and viruses have been evidenced. First, viruses directly utilize CLDNs as bind/entry factors to initiate infection; e.g., CLDN1 acts as a co-receptor for hepatitis C virus (HCV) [31,32]. Second, more instances have been observed of a virus evoking the aberrant distribution or degradation of CLDNs to destroy the permeability of TJs. Here, CLDNs exert an indirect effect to accelerate viral dissemination. This perspective can be supported by the West Nile virus (WNV)-induced degradation of CLDN1~4 [30], HIV or Enterovirus A71 virus-altered expression of CLDNs and the permeability of the cell barrier [33,34]. A similar phenomenon was viewed in this work, where expression of CLDN3 was impeded by LMIV infection. Furthermore, hindered expression of CLDN3 corresponded to the impaired TJs of virus-infected LMB-L cells, which was proved by qRT-PCR and in vitro FITC-Dextran permeability assay, respectively. LMB-L cells were identified as astroglia cells in our previous study [5]. Given a lack of astrocytes in teleost, astroglia undertakes the same functions as astrocytes involving the maintenance of barrier structure integrity [35]. Thus, LMIV causes the collapse of TJs in LMB-L cells, possibly leading to the dysfunction of barrier structure integrity of spotted sea bass.

After we verified the interaction between CLDN3 expression and LMIV replication, we continually sought to interpret how LMIV suppressed the expression of CLDN3. Our pilot investigation on miRNA-mRNA joint analysis inspired us to attribute the negative regulation of CLDN3 mRNA to miRNA-181a. MiR-181a was activated by LMIV infection, presenting a 4.83-fold up-regulation. Furthermore, the targeted regulation of miR-181a on CLDN3 was confirmed by dual-luciferase reporter assays. Then, taking into account the negative correlation of CLDN3 and LMIV, LMIV infection stimulated the expression of miR-181a, then miR-181a promoted the degradation of CLDN3 mRNA, in turn, down-regulated CLDN3 contributed to LMIV replication. The miRNA-mRNA regulatory relationship expands our understanding of the virus–host interaction. Several teleost viruses have been described as advancing efficient multiplication by virtue of this tactic. Grouper miR-122 reduces the expression of inflammatory genes by binding to p38 α MAPK to enhance the proliferation of Singapore grouper iridovirus (SGIV) [36]. MiR-124 blocks CyHV-2-induced apoptosis by targeting ccBax to improve the virus replication in in vitro assays [37]. Conversely, microRNAs are also capable of inhibiting virus multiplication, such as miR-155 and miR-722 negatively regulating CyHV-3 by interfering with the host’s innate immune system and virus-encoded genes, respectively [38,39]. Thus, miRNAs provide a novel perspective for better understanding the interaction between host and virus.

## 5. Conclusions

In conclusion, CLDN3 was degraded by miR-181a, which was activated by LMIV infection; furthermore, down-regulation of CLDN3 promoted LMIV proliferation and might be responsible for the dysfunction of TJs, leading to the collapse of the physical defense against pathogens. A new “miR-181a/CLDN3/LMIV” regulatory relationship has been confirmed by this work, which sheds new light on the mechanisms of LMIV replication. However, the in-depth molecular mechanisms by which LMIV regulates the expression of miR-181a and CLDN3 requires further studies.

## Figures and Tables

**Table 1:** Primers and cloning sites used in this study.

Name	Primer Sequence (5′ → 3′)	Source	Fragment Length (bp)	Annealing Temperature (°C)	Use
Primers for Claudin-3 and RNA polymerase II amplification and expression					
CLDN3-F	ATGTCTATAGGGCTGGAGTTGAT	URX57470.1	648	55	Clone of Claudin-3 ORF
CLDN3-R	TCATACATAGTCTTTCCTTTC				
CLDN3-pET28a-F	GCGAATTCAGCACGGGGCAGATGCAGTGTAAG	This study	192	58	Clone of Claudin-3-pET28a
CLDN3-pET28a-R	GCCTCGAGTACATAGTCTTTCCTTTCTAAC				
CLDN3-pcDNA3.1-F	GGGCTAGCATGGCTATAGGGCTGGAGTTGATAG	This study	645	58	Clone of Claudin-3-pcDNA3.1 (+)
CLDN3-pcDNA3.1-R	CCGCTCGAGTACATAGTCTTTCCTTTC				
RNA poly II-F	ATGCCGTATGCTAACCAA	This study	828	55	Clone of RNA polymerase II ORF
RNA poly II-R	TCAGTTGATGGTGAGCACGTC				
RNA poly II-pET28a-F	CGGAATTCCCTCTCACAAGCGACGACATC	This study	459	55	Clone of RNA polymerase II-pET28a
RNA poly II-pET28a-R	CCGCTCGAGTCCGTTGGGGTCATAGGGAG				
Primers for Claudin-3 3′UTR clone for determine the interaction between miR181a and 3′-UTR of CLDN3					
CLDN3 3′-UTR-F	CCGCTCGAGACGCTAATGTTATCGTCA	This study	372	62	Clone of pmirGLO-CLDN3-3′-UTR and pmiGLO-CLDN3-3′-UTR-Mut
CLDN3 3′UTR-R	ACGCGTCGACTGGTGCTTTTGTGCTGCC				
CLDN3 3′-UTR-mut-F	ACAAAATTGAAGAAAACCTACAGTTAAAAGGC	This study	372	62	Clone of pmiGLO-CLDN3-3′-UTR-Mut
CLDN3 3′UTR-mut-R	TTCTTCAATTTTGTACACGTGTACAAAATTGAAGAA				
Primers for real-time PCR					
CLDN3F (qPCR)	AGGGCCTGTGGATGACTT	This study	123	60	Claudin-3 qRT-PCR
CLDN3R (qPCR)	GGATGGAGATAACGGTGAG				
LMIV MCP-F (qPCR)	ATCAGCCAGAGCACCCAG	This study	146	60	LMIV qRT-PCR
LMIV MCP-R (qPCR)	CTCACGCTCCTCACTTGTC				
RNAploy II-F (qPCR)	GTCAGGAACTACGGCTCAGG	This study	117	60	RNA polymerase II qRT-PCR
RNAploy II-R (qPCR)	TGTGCCTCAGTGCATTGTCT				
miR181a-F (qPCR)	AACATTCAACGCTGTCGGTGAG	This study	22	60	miR181a qRT-PCR
miR181a-R (qPCR)	AAGCAGTGGTATCAACGCAGAGTAC				
U6-F (qPCR)	TTTGGAACGCTTCACGAATTTGC	This study	70	60	U6 gene qRT-PCR
U6-R (qPCR)	GGAACGATACAGAGAAGATTAGCA				

**Table 2:** CLDN3 siRNA sequence.

Name	Sense (5′ → 3′)	Anti-Sense (5′ → 3′)
CLDN3-176	GGCAGAUGCAGUGUAAGGUdTdT	ACCUUACACUGCAUCUGCCdTdT
CLDN3-623	GGUUAGAAAGGAAAGACUAdTdT	UAGUCUUUCCUUUCUAACCdTdT
siRNA-control	UUCUCCGAACGUGUCACGUdTdT	ACGUGACACGUUCGGAGAAdTdT

## Data Availability

The authors declare that all data supporting the findings of this study are available within the paper.

## Supplementary Materials

The following supporting information can be downloaded at: https://www.mdpi.com/article/10.3390/v16101589/s1, Figure S1: Expression determination of CLDN3 by Western blotting and indirect immunofluorescence. (a) SDS-PAGE and Western blotting analysis of recombinant spotted sea bass CLDN3. Lane M—protein molecular mass marker; Lane 1—pET28a-CLDN3/*E. coli* Rosetta after induction with IPTG; Lane 2—pET28a/*E. coli* Rosetta after induction with IPTG; Lane 3—purified CLDN3 (13.0 kDa); Lane 4—Western blotting of purified CLDN3 (13.0 kDa). (b) SDS-PAGE and Western blotting analysis of recombinant spotted sea bass RNA polymerase II. Lane M—protein molecular mass marker; Lane 1, pET28a/*E. coli* Rosetta after induction with IPTG; Lane 2—pET28a-RNA polymerase II/*E. coli* induction with IPTG; Lane 3—purified RNA polymerase II (26.0 kDa); Lane 4—Western blotting of purified RNA polymerase II (26.0 kDa).
