# Supplementary material for: MiR-181a Negatively Regulates Claudin-3 to Facilitate Lateolabrax maculatus Iridovirus Replication in Lateolabrax maculatus Astroglia Cells
Source: Viruses. 2024 Oct 9;16(10):1589. doi: 10.3390/v16101589 (PMC11512373; doi:10.3390/v16101589)
Supplement: Supplementary file 1 [file viruses-16-01589-s001.zip › viruses-3182269-supplementary.pdf]

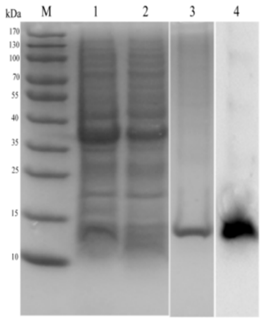

(a)

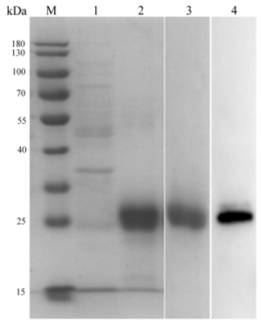

(b)

Figure S1. Expression determination of CLDN3 by Western blotting and indirect immunofluorescence. (a) SDS-PAGE and Western blotting analysis of recombinant spotted sea bass CLDN3. Lane M—protein molecular mass marker; Lane 1—pET28a-CLDN3/*E. coli* Rosetta after induction with IPTG; Lane 2—pET28a/*E. coli* Rosetta after induction with IPTG; Lane 3—purified CLDN3 (13.0 kDa); Lane 4—Western blotting of purified CLDN3 (13.0 kDa). (b) SDS-PAGE and Western blotting analysis of recombinant spotted sea bass RNA polymerase II. Lane M—protein molecular mass marker; Lane 1, pET28a/*E. coli* Rosetta after induction with IPTG; Lane 2—pET28a-RNA polymerase II/*E. coli* induction with IPTG; Lane 3—purified RNA polymerase II (26.0 kDa); Lane 4—Western blotting of purified RNA polymerase II (26.0 kDa).
